# Rapid synthesis of transition metal dichalcogenide–carbon aerogel composites for supercapacitor electrodes

**DOI:** 10.1038/micronano.2017.32

**Published:** 2017-07-17

**Authors:** Matthew J. Crane, Matthew B. Lim, Xuezhe Zhou, Peter J. Pauzauskie

**Affiliations:** 1 Department of Chemical Engineering, University of Washington, Seattle, WA 98195-1750, USA; 2 Department of Materials Science & Engineering, University of Washington, Seattle, WA 98195-2120, USA; 3 Fundamental & Computational Sciences Directorate, Pacific Northwest National Laboratory, Richland, WA 99352, USA

**Keywords:** aerogel, molybdenum disulfide, nanomanufacturing, rapid, resorcinol-formaldehyde, supercapacitor, transition metal dichalcogenide, tungsten disulfide

## Abstract

Transition metal dichalcogenide (TMD) materials have recently demonstrated exceptional supercapacitor properties after conversion to a metallic phase, which increases the conductivity of the network. However, freestanding, exfoliated transition metal dichalcogenide films exhibit surface areas far below their theoretical maximum (1.2 %), can fail during electrochemical operation due to poor mechanical properties, and often require pyrophoric chemicals to process. On the other hand, pyrolyzed carbon aerogels exhibit extraordinary specific surface areas for double layer capacitance, high conductivity, and a strong mechanical network of covalent chemical bonds. In this paper, we demonstrate the scalable, rapid nanomanufacturing of TMD (MoS_2_ and WS_2_) and carbon aerogel composites, favoring liquid-phase exfoliation to avoid pyrophoric chemicals. The aerogel matrix support enhances conductivity of the composite and the synthesis can complete in 30 min. We find that the addition of transition metal dichalcogenides does not impact the structure of the aerogel, which maintains a high specific surface area up to 620 m^2^ g^−1^ with peak pore radii of 10 nm. While supercapacitor tests of the aerogels yield capacitances around 80 F g^−1^ at the lowest applied currents, the aerogels loaded with TMD’s exhibit volumetric capacitances up to 127% greater than the unloaded aerogels. In addition, the WS_2_ aerogels show excellent cycling stability with no capacitance loss over 2000 cycles, as well as markedly better rate capability and lower charge transfer resistance compared to their MoS_2_-loaded counterparts. We hypothesize that these differences in performance stem from differences in contact resistance and in the favorability of ion adsorption on the chalcogenides.

## Introduction

In response to rapid improvements in renewable energy generation, electrochemical supercapacitors with high power densities and rapid cycling have emerged as a promising technology to bridge the energy density storage and variable energy density demands of grid management and hybrid vehicles^1^. State of the art batteries employ lithium intercalation that enables 3–30 times more charge storage than supercapacitors. However, supercapacitors can provide 2–3 orders of magnitude greater power^2^. While typical lithium-ion batteries store electrochemical potential via phase transformative redox reactions, supercapacitors do so by means of an electrical double layer in a working electrolyte and/or rapid charge transfer processes with no concomitant phase transition, that occurs in response to an applied voltage^2,3,4,5^. Thus, supercapacitors do not face the performance-limiting factors of reaction kinetics, ion transport through bulk electrode material, and accompanying volume changes that are characteristic of batteries. Improvements in efficient, scalable, and economical syntheses are needed to drive supercapacitor applications.

Because double-layer formation dictates power densities, nanostructured materials, such as pyrolyzed carbon aerogels^6,7,8,9^, with high specific surface areas (SSA) have emerged as the premier supercapacitors^10^. Carbon aerogels are amorphous, sp^2^- and sp^3^-bonded carbon structures formed from the reaction of resorcinol and formaldehyde with high SSAs (>500 m^2^ g^−1^), narrow pore sizes, and low densities^11,12,13,14^. Upon pyrolysis, these aerogels exhibit improved electrical conductivity (up to 50 S cm^−1^) and SSA with benign chemistry, making them an intriguing material for supercapacitors^14,15^. To improve their performance, research groups have attempted to load them with high-conductivity or high-capacitance materials^16,17^. However, their lengthy synthesis time (typically 24 h or longer) prevents economical scalability, limiting their widespread use^6,12^.

Transition metal dichalcogenides (TMDs) are layered, graphite-like van der Waals structures composed of a transition metal layer sandwiched between two chalcogenide layers that have shown promise as supercapacitor active materials due to the range of oxidation states available to transition metals^18,19,20,21^. A recent report demonstrated that MoS_2_ capacitance significantly improves after conversion from the semiconducting 2H phase to the metallic 1T phase, due to enhanced electrical conductivity—the in-plane conductivity of 2H MoS_2_ is ~0.2 S cm^−1^ and that of the 1T phase is 10–100 S cm^−1^—and increased ion intercalation mobility^19,22,23^. Similarly, 2H MoS_2_ and WS_2_-based supercapacitor performance improved after the creation of a mesoporous with enhanced conductivity^24,25^. However, it is important to note that the most successful 1T devices suffered from a low SSA of 9 m^2^ g^−1^, and required the use of pyrophoric n-butyllithium to induce a phase change, both of which mitigate the scalability of their impressive performance. In addition, freestanding TMD films do not exhibit mechanical stability in electrochemical environments, which sets a limit to the maximum size of unsupported films and inhibits high surface area applications^26^.

To address the aforementioned limitations, we present in this work a synthesis that emphasizes scalable processes to encapsulate exfoliated TMD sheets in an aerogel matrix via benign and ultrafast resorcinol-formaldehyde (RF) sol-gel chemistry^6,11,13,16^. This process employs the ultrasonication of TMD precursors—MoS_2_ and WS_2_—in acetonitrile to produce exfoliated 2H MoS_2_ and WS_2_, respectively, followed by the rapid, acid-catalyzed gelation of RF within 30 min at mild temperatures. After supercritical drying and high-temperature pyrolysis, the resulting carbon aerogel provides a high surface area, mechanically stable, and electrically conductive support for TMDs that is well suited for electrochemical devices. To demonstrate these applications, we perform supercapacitor tests on our TMD-loaded carbon aerogels that yield favorable specific capacitances around 80 F g^−1^, volumetric capacitances approaching 60 F cm^−3^, and low operational electrochemical impedance.

## Materials and methods

When exfoliated TMD sheets dry, they restack to form low surface area (9 m^2^ g^−1^) films^19,27^. As such, we designed the rapid synthesis to avoid allowing the TMD solution to dry before the gelation can trap any exfoliated sheets. In addition, while we employ sonication^28,29^ to disperse the TMDs, there are a range of other potentially scalable exfoliation methods including shear mixing^30^, direct synthesis^31^, and intercalation^32,33^ to name a few.

**Figure 1:**
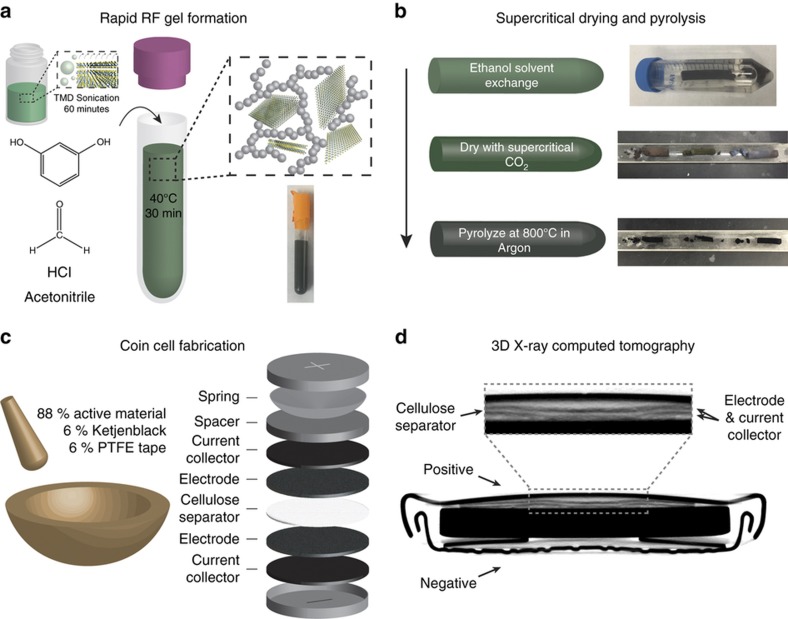
Outline of the nanomanufacturing process for composite TMD carbon aerogels. Initially, sonication-driven cavitation (**a**) drives TMD sheets apart and enhances dispersion within acetonitrile. Resorcinol and formaldehyde are added to this solution, which causes rapid sol-gel formation, catalyzed by hydrochloric acid. This gel is washed with ethanol to remove any unreacted species and dried with supercritical CO_2_ (**b**) to displace the solvent without destroying its pore structure before being pyrolyzed in argon. Finally, we process this material into a supercapacitor by grinding it with carbon black additive (Ketjenblack) and PTFE tape, rolling and punching it into electrodes that are assembled into a symmetric coin cell, and adding the resulting electrode to a full coin cell, using a cellulose separator, illustrated in (**c**). A three-dimensional X-ray computed tomography image of a coin cell after 10 000 charge–discharge cycles (**d**).

Figure 1 outlines the TMD aerogel synthetic scheme. In the first step, a temperature-controlled bath sonicator (22 °C; Branson 1510R-DTH, Danbury CT, USA) exfoliates and disperses TMD sheets in acetonitrile (ACN, EMD Millipore, Billerica MA, USA) at concentrations from 8.6 to 34 mg mL^−1^. However, we were able to synthesize aerogels at TMD loadings of up to 100 mg mL^−1^ in acetonitrile. After sonication for 60 min, we transferred this solution to a polypropylene tube and added resorcinol (R, Sigma-Aldrich, St. Louis, MO, USA), formaldehyde (F, 37 wt-% methanol-stabilized aqueous solution, Sigma-Aldrich), and hydrochloric acid (C, 37 wt-%, Macron, Center Valley PA, USA) to achieve molar ratios of R:F=1:2, R:C=8.4:1, and R:ACN=1:76^6,12^, which result in a 2:1 ratio by weight of resorcinol to TMD for a 17.1 mg mL^−1^ starting dispersion of TMD. This corresponds to molar ratios for resorcinol to MoS_2_ (powder, Sigma-Aldrich) and WS_2_ (powder, Alfa Aesar, Haverhill MA, USA) of 2.9:1 and 4.5:1, respectively. For WS_2_-loaded gels, we prepared additional samples by the same technique using initial WS_2_ dispersions of 8.6 and 34 mg mL^−1^, corresponding respectively to 4:1 and 1:1 weight ratios, or 9.0:1 and 2.25:1 molar ratios, of resorcinol to WS_2_. We also prepared a control sample with no TMD. The mixture of reagents was quickly placed in the bath sonicator set to 40 °C for 30 min. During this time, the resorcinol undergoes electrophilic aromatic substitution at the 2, 4, and 6 positions with formaldehyde to form methylene and methylene-ether bridges^34^. We then washed the aerogel with ethanol three times over 36 h to remove the acetonitrile and dried it with supercritical CO_2_ in an autoclave (E3100, Quorum Technologies, Laughton, East Sussex, UK). Because it has low density and surface tension, supercritical CO_2_ displaces the ethanol and preserves pore structure to produce a high surface area product. Finally, we pyrolyzed the aerogels in a tube furnace at 800 °C in an argon atmosphere for 4 h, which drives off oxygen moieties, yielding a high conductivity sp^2^- and sp^3^-bonded support of carbon spheres. We found that annealing at 1000 °C destroyed the TMDs. In addition, we note that this pyrolysis step mimics the current industrial synthesis of supercapacitors^35^. Before pyrolysis, the MoS_2_ and WS_2_ loaded aerogels exhibit a dull, deep blue, and green color, respectively, whereas the pure RF aerogel has a brick-red color. Following pyrolysis, the aerogels all exhibit a dark black color, indicative of carbonization (Figure 1b)^16^. Henceforth, the pyrolyzed pure RF aerogel will be abbreviated RFA, the pyrolyzed MoS_2_-loaded aerogel will be abbreviated MA-17, and the pyrolyzed WS_2_-loaded aerogel will be abbreviated WA-8.6, WA-17, or WA-34 according to the concentration of the initial TMD dispersion. Compared with the chemical exfoliation of TMD’s via pyrophoric n-butyllithium^19^ and the long gelation times in other syntheses^6,12^, our sol-gel synthesis represents a rapid, mild, and benign process.

To characterize the aerogels, we employed nitrogen adsorption, X-ray diffraction (XRD), Raman spectroscopy, Fourier transform infrared (FTIR) transmission spectroscopy, and transmission electron microscopy (TEM). We collected nitrogen sorption isotherms using a NOVA 2200e porosimeter (Quantachrome, Boynton Beach, FL, USA), heating samples in vacuum at 200 °C for at least 12 h prior to analysis to drive off pre-adsorbed species. From the isotherms, we obtained surface area and pore size distributions with Brunauer–Emmett–Teller (BET) theory and Barrett–Joyner–Halenda (BJH) theory, respectively. Specifically, multipoint surface area calculations used data from the relative pressure (*P*/*P*_0_) range between 0.05 and 0.30, and the pore size distributions used the desorption isotherm. We performed Raman spectra using a home built setup comprised of a 532 nm laser (Coherent Compass, Santa Clara, CA, USA) focused with a 50x objective (0.55 numerical aperture, Mitutoyo, Kawasaki, Kanagawa, Japan) and collected on a spectrometer fitted with a liquid nitrogen-cooled CCD detector (SpectraPro 500i, Acton Research Corporation, Acton MA, USA). To collect the XRD data, we used a Bruker (Billerica MA, USA) D8 Discover X-ray diffractometer equipped with a General Area Detector Diffraction System (GADDS) and a Cu *K*_α_ source at 1.54 Å. FTIR measurements were performed by the KBr pellet method with a Bruker VERTEX 70 spectrometer in transmission mode. Finally, bright-field TEM images with accompanying selected-area electron diffraction (SAED) patterns and energy-dispersive X-ray spectra (EDX) were obtained on an FEI (Hillsboro, OR, USA) Tecnai G2 F20 with 200 kV accelerating voltage.

In addition to MoS_2_ and WS_2_, we attempted to synthesize a selenium-based TMD aerogel composite, by adding NbSe_2_. However, after pyrolysis, both Raman and XRD demonstrated that the NbSe_2_ had oxidized into Nb_2_O_5_ and NbO_2_, as shown in Supplementary Figure S8.

To fabricate coin cell electrodes, we ground a mixture of pyrolyzed TMD-loaded aerogels in a rotary mill (Fritsch Pulverisette, Idar-Oberstein, Germany) and passed it through a #140 test sieve (opening size ~106 μm). We ground the milled, sieved product (88 wt%) with PTFE tape (6 wt%) as a binder, and Ketjenblack carbon additive (6 wt%, AkzoNobel, Arnhem, the Netherlands) together with a mortar and pestle until the mixture was completely amalgamated. We then flattened the amalgam with a glass rolling pin to ~100 μm thick sheet, from which we punched 0.5 inch diameter electrodes. We assembled supercapacitor devices in a symmetric two-electrode configuration consisting of a sandwich of SUPER-P carbon black (Timcal, Bironico, Switzerland) @ aluminum foil current collectors, CR2032 coin cell casings (MTI, Richmond CA, USA) and TMD-loaded aerogel electrodes surrounding a cellulose separator, filled with 1 M Na_2_SO_4_ aqueous electrolyte (Figure 1c).

The electrochemical performance of our supercapacitor cells was evaluated using a Bio-Logic (Seyssinet-Pariset, France) VMP3 potentiostat/galvanostat with EC-Lab software. We first conditioned the cells over 5 galvanostatic charge–discharge cycles between 0.1 and 0.9 V, one cycle at 1 mA and four at 10 mA, to ensure complete electrolyte permeation. This was followed by six more galvanostatic cycles between 0.1 and 0.9 V, which were used for capacitance measurements. In these cycles, the cell was charged at 10 mA and discharged at increasing gravimetric current densities of 0.054, 0.28, 0.56, 1.39, 2.78, and 5.53 A g^−1^ for MA-17; and 0.059, 0.30, 0.60, 1.51, 3.02, and 6.03 A g^−1^ for all WS_2_ aerogels as well as the RFA (normalized to the mass of active material). For the samples made from 17 mg mL^−1^ TMD dispersions, this corresponds to currents of 1, 5, 10, 25, 50, and 100 mA. Immediately after rate testing, we conducted electrochemical impedance spectroscopy (EIS) at 0.5 V with a 5 mV sinusoidal oscillation between 400 kHz and 10 mHz. Finally, we analyzed the coin cells with cyclic voltammetry (CV), sweeping between 0 and 0.9 V at 20 mV s^−1^. For WA-17, a galvanostatic cycling test was performed following cyclic voltammetry, whereby the cell underwent continuous charge–discharge cycles between 0.1 and 0.9 V at a fixed current density of 0.25 A g^−1^.

## Results and discussion

**Figure 2:**
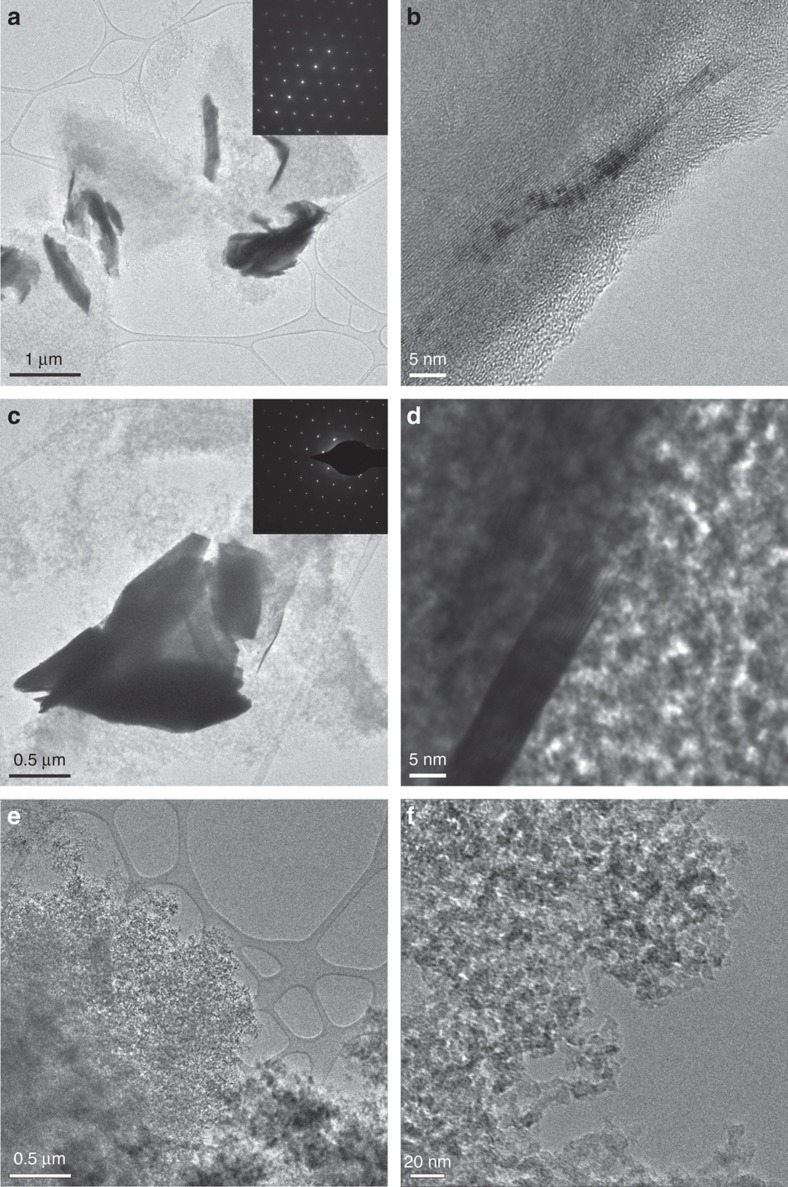
TEM images of MA-17 (**a** and **b**), WA-17 (**c** and **d**), and RFA (**e** and **f**). **e** and **f** are characteristics of the gels as synthesized, while (**a**–**d**) demonstrate the presence of exfoliated sheets. Insets in the TEM images **a** and **c** show electron diffraction of the TMD sheets dispersed in the aerogel.

TEM images of the pyrolyzed aerogels in Figure 2 confirmed that the RF matrix, which consisted of nanoscale carbon particles characteristic of a pyrolyzed aerogel, acted as a support for the TMD sheets. Electron diffraction (Figures 2a and b, inset) and energy-dispersive X-ray spectroscopy (Supplementary Figures S9 and S10) of the doped aerogels demonstrate that the sol-gel process does not chemically modify the TMDs. After incorporation, these TMD crystals range in size from 5–100 nm in the (002) stacking plane and up to micron scale in length. While it does not have a high exfoliation efficiency, acetonitrile is effective at preserving large area sheet sizes by physisorbing to the chalcogenide atoms in TMDs to reduce van der Waals forces before subsequent intercalation^36^. This mild reduction is believed to prevent scissoring of TMDs and lead to large area sheet dispersion (Figures 2b and d)^37^. The WS_2_ composite exhibited the highest degree of exfoliation, as evidenced by additional TEM images in Supplementary Figure S1.

**Figure 3:**
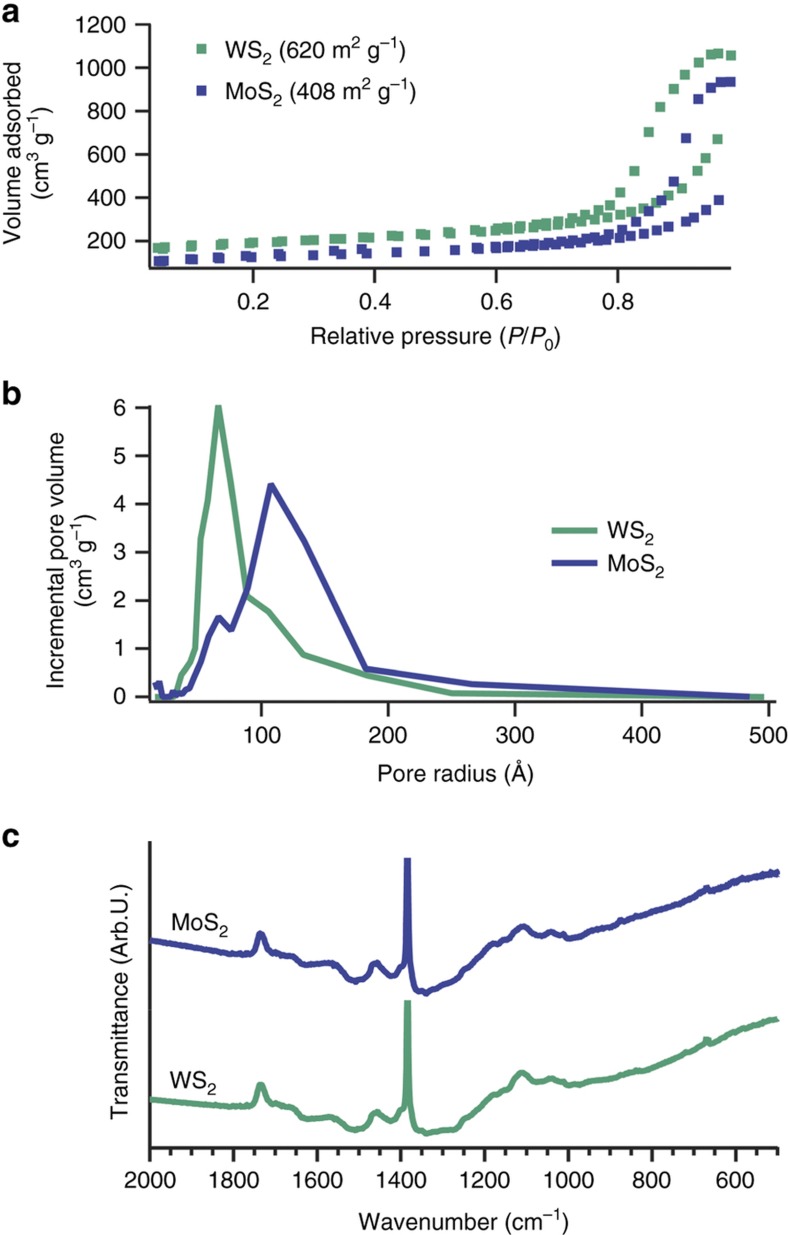
Nitrogen sorption isotherms with BET surface area (**a**) and BJH pore size distribution (**b**) of MA-17 and WA-17. In addition, FTIR transmittance data (**c**) demonstrate functional groups within the aerogel composites.

BET analysis of the aerogels (Figure 3, Supplementary Figure S2 and Table 1) demonstrated that the neither the addition of TMD sheets in this accelerated synthesis, nor the amount of TMD added significantly impacted the surface area or the morphology of the gel. All the TMD-loaded aerogels maintained high surface areas greater than 400 m^2^ g^−1^, with a maximum for WA-17 at 620 m^2^ g^−1^. Furthermore, processing the aerogels for supercapacitor electrodes did not significantly affect their surface area: WA-17 retained 94% of its original surface area after milling and sieving, and electrode sheets made from combining the same milled and sieved sample with PTFE tape and carbon black retained 99% of the original surface area of the aerogel (Supplementary Figure S7). These results suggest that the aerogel represents a mechanically stable support throughout processing.

Comparing the theoretical maximum surface areas for the TMD’s (750 and 483 m^2^ g^−1^, see Supplementary Information Section 1) to our control aerogel of pure pyrolyzed RF (776 m^2^ g^−1^), it is clear that the carbon aerogel constituted the majority of the surface area^6^. In addition, the nitrogen sorption isotherms of all the aerogels, including the unloaded control sample, exhibited type H1 hysteresis^38^, which is characteristic of largely uniform diameter spherical particles. This further confirms that the carbonaceous matrix constituted the bulk of the surface area in the TMD-loaded samples. Indeed, the TEM images in Figure 2 show that the TMD’s incorporated as large sheets of material supported by the homogeneous network of carbonized RF polymer. The BJH pore size distributions of the TMD-loaded aerogels were roughly unimodal and peaked below 100 Å pore radius, revealing their mesoporous nature. In contrast, the RFA featured a bimodal pore distribution with larger pores on average than the other samples, as evidenced by the peaks at 90 and 135 Å, and a tail extending past 250 Å.

In FTIR spectra of MA-17 and WA-17 (Figure 3c), the absence of epoxy functional groups at 1220 cm^−1^ and alkoxy groups at 1095 cm^−1^, which form during polycondensation of resorcinol and formaldehyde, shows that pyrolysis successfully removed these oxygen-containing moieties^39^. The broad band centered at 1510 cm^−1^ along with the weaker band at 1630 cm^−1^ correspond to C=C–C stretching in an aromatic ring and is evidence of carbonized sp^2^-bonded structures in the aerogels. The band at 1340 cm^−1^ represents O–H bending in phenol groups, which have been observed to survive heat treatment even at 1000 °C, well above the pyrolysis temperature of 800 °C for our aerogels^40^. Notably, both aerogels exhibit a small peak at 680 cm^−1^, corresponding to a C–S mode, which suggests that the rapid synthesis and subsequent pyrolysis produces chemical bonding between the RF matrix and the TMD sheets.

**Figure 4:**
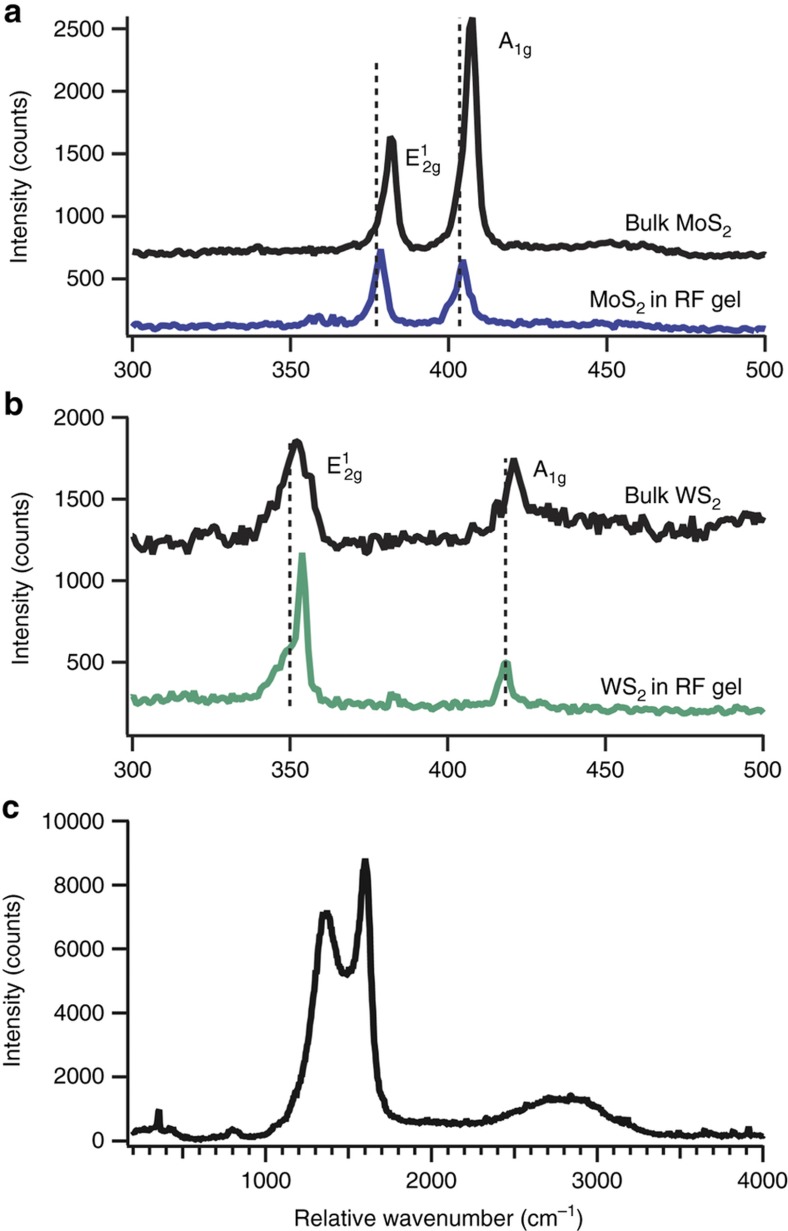
Raman characterization of MoS_2_ (**a**) and WS_2_ (**b**) dispersed within the RF matrix of the aerogel (**c**) for samples synthesized from 17 mg mL^−1^ TMD dispersions. The Raman scattering of the aerogel (**c**) was collected from the WS_2_ composite. For each RF-supported TMD, the bulk Raman spectra is displayed offset for comparison. In addition, the supported TMD and bulk Raman spectra were collected without adjusting the spectrometer grating to prevent alignment-induced shifts in wavenumber. All wavenumbers were further calibrated with a silicon wafer. Vertical dotted lines represent peak centers of the in-plane (E^1^_2g_) and out of plane (A_1g_) modes of the exfoliated TMD’s to emphasize the shift from their bulk counterparts due to exfoliation in the case of MoS_2_ and WS_2_.

The Raman spectrum of WA-17 shows both the D band at 1345 cm^−1^ and the G band at 1603 cm^−1^ (Figure 4c). The D band stems from carbon-carbon sp^3^ stretching with A_1g_ symmetry, associated with disordered atoms, while the G band originates from the doubly degenerate (iTO and LO phonon modes) carbon stretching with E_2g_ symmetry. Interestingly, the intensity ratio of these modes and the location of the G band provide information about both the amount of sp^3^ bonding and the graphitic grain size domain. As the G band decreases in wavenumber and the intensity ratio of the D band to G band decreases, carbon bonding shifts from graphite to nanocrystalline graphite to amorphous carbon^41^. This analysis suggests that these aerogels contain approximately 5% sp^3^ bonding with graphitic grain sizes of 11 nm. Prior reports have shown that the addition of transition metal ions into a carbon aerogel can catalyze graphitization during pyrolysis at temperatures greater than 1000 °C (Ref. 42). However, we do not observe any catalytic graphitization of the aerogel from Raman spectroscopy.

All the TMD’s exhibit E^1^_2g_ and A_1g_ symmetry Raman active modes, which correspond to in-plane and out-of-plane stretching modes, respectively^43^. Similar to carbon, the distance and intensity ratio between these scattering modes gives information about the degree of electrical coupling between layers. The addition of more monolayers tends to increase the energy of the out-of-plane A_1g_ mode. While the TEM data do not suggest high exfoliation of the TMD’s in the gels, the shift between peaks implies that there is a decrease in interlayer coupling, which could lead to increased adsorption or intercalation of ions during supercapacitor operation. For WS_2_, the spacing between the E^1^_2g_ and A_1g_ peaks decreases from 69 cm^−1^ to 64 cm^−1^ after sonication, which implies that monolayers are electrically coupled to two nearby sheets (Figure 4b)^43,44^. We note that this does not necessarily mean that the sheets were highly exfoliated, only that interlayer coupling in the (001) direction decreased during processing. For MoS_2_, there is a less distinct shift, which suggests that the exfoliated material is only slightly shifted from its bulk counterpart (Figure 4a). The exfoliated, dispersed (bulk) E^1^_2g_ peak sits at 378 cm^−1^ (381 cm^−1^) and the A_1g_ sits at 404 cm^−1^ (407 cm^−1^), leading to a difference of 26 cm^−1^ (26 cm^−1^). A comparison to literature for the out-of-plane A_1g_ shows that each MoS_2_ remained coupled to only one other layer. However, the E^1^_2g_ and peak spacing suggest the material retained its bulk-like characteristics^43,45^.

**Figure 5:**
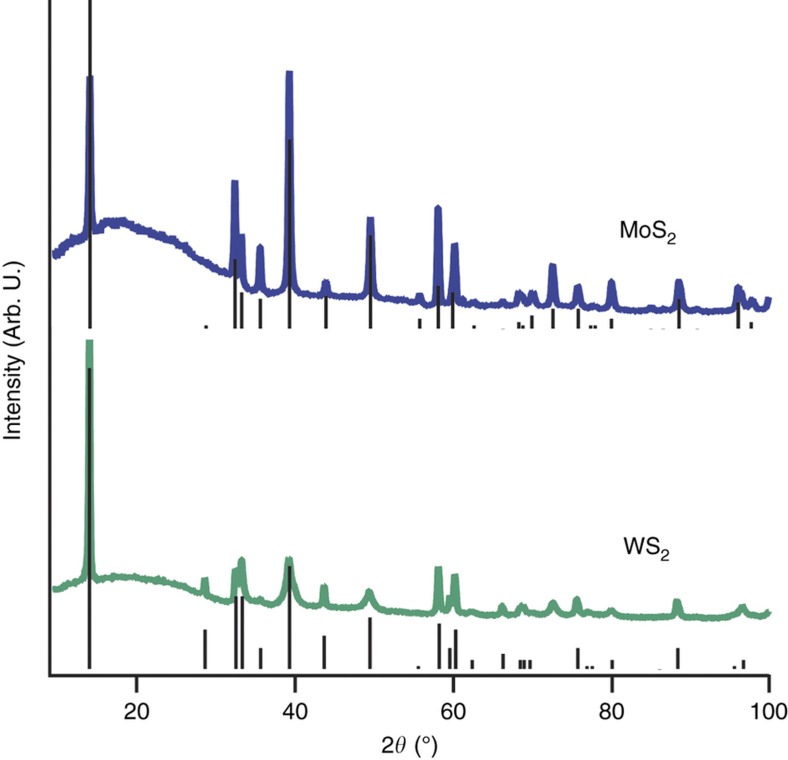
XRD patterns of MA-17 and WA-17. Vertical solid lines show the corresponding X-ray diffraction (XRD) peaks and relative intensities of 2H-MoS_2_ and 2H-WS_2_ from the International Centre for Diffraction Data cards.

The XRD and SAED of both MoS_2_ and WS_2_-loaded aerogels demonstrated that the TMD’s remained crystalline throughout the rapid sol-gel processing and the subsequent high-temperature pyrolysis (Figures 2 and 5). For these sulfur-based TMD’s, we identified the sharp peaks in XRD as highly crystalline 2H phases. The underlying broad peak centered at 2*θ*=17° originates from amorphous carbon within the aerogel^6^. By examining the peak broadening, we further quantified the size of the TMD crystals loaded into the aerogels, using the Scherrer equation (Supplementary Information, Section 2). This analysis suggested that on average, the thickness of the WS_2_ crystallites in the (002) axis is about the same for all the WS_2_-loaded aerogels, around 100 nm or 160 layers; whereas the thickness of the MoS_2_ crystallites in the MoS_2_-loaded aerogel is somewhat lower at 64 nm or 104 layers (Table 1). This agrees well with the cross-sectional TEM images of the pyrolyzed aerogels (Figure 2).

**Figure 6:**
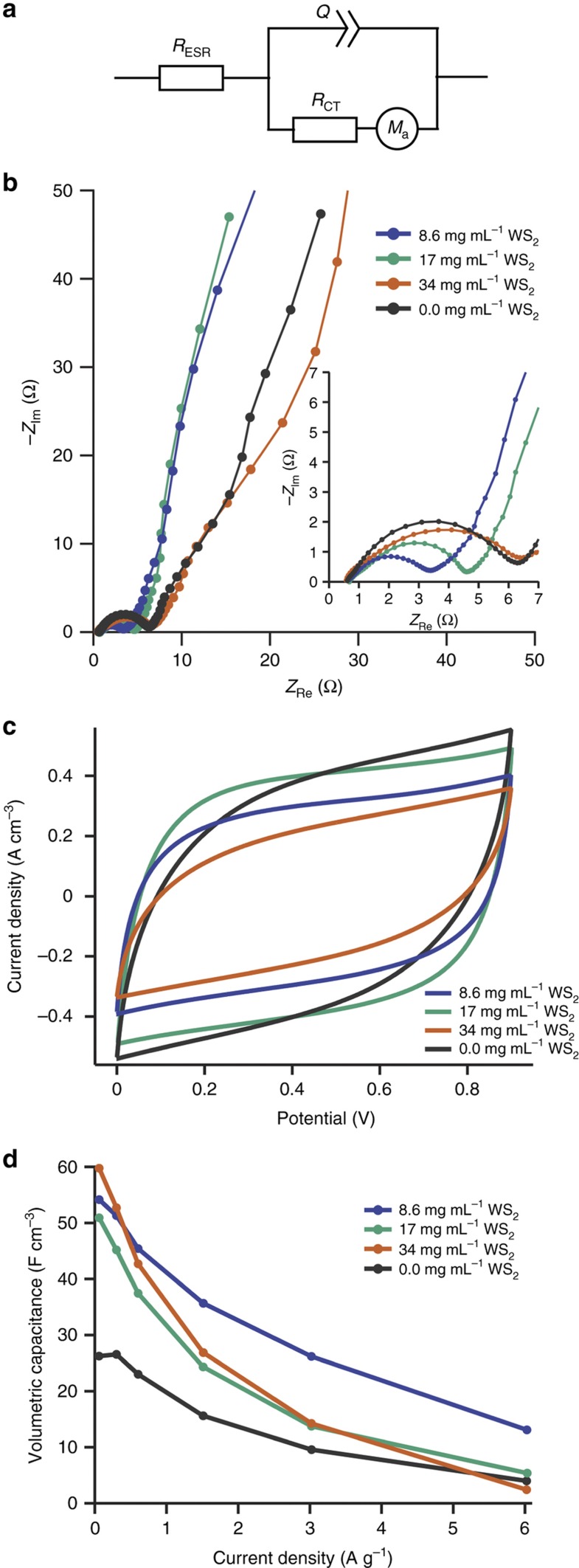
Electrochemical characterization of the pyrolyzed aerogels with different WS_2_ mass loadings fabricated into coin cell supercapacitors, including an equivalent circuit diagram (**a**), Nyquist plots from EIS (**b**, inset shows more detail of high-mid frequency range), cyclic voltammogram at sweep rate 20 mV s^−1^ (**c**), and specific volumetric capacitance (**d**) as a function of applied current density from galvanostatic tests.

From electrochemical tests of our pyrolyzed TMD aerogel supercapacitor electrodes, we evaluated specific volumetric capacitance based on galvanostatic discharge profiles at each applied current, using the full voltage window of 0.9–0.1 V (Supplementary Information, Section 3 and Figure 6d). As we vary the mass loading of TMD’s into the aerogel, we observed significant differences in the densities of the aerogels: 0.33, 0.61, and 0.90 g cm^−3^ for RFA, MA-17, and WA-8.6, respectively. The wide range reflects the significant differences in the TMD densities—7.5 g cm^−3^ for WS_2_ and 5.06 g cm^−3^ for MoS_2_—as well as their molecular weights. For example, while the WS_2_ comprises only 3.3 mol% of WA-17, it represents 41.5 mass-%. Thus, the resulting capacitances represent the interplay between the molar percentage of the TMD and the density, capacitance, surface area, and conductivity of the added TMD, as discussed below.

While undoped and doped aerogels exhibited similar gravimetric capacitances (87.5 and 84.5 F g^−1^ maxima, respectively), the volumetric capacitance increased significantly upon the addition of TMD’s (Table 1). The WA-34 exhibited the greatest volumetric capacitance of the samples at 59.8 F cm^−3^ (64.7 F g^−1^), 127% greater than RFA at 26.3 F cm^−3^ (87.5 F g^−1^). Similarly, the MA-17 featured a large volumetric capacitance compared to the RFA, at 52.5 F cm^−3^ (84.5 F g^−1^), as did the other WS_2_-loaded aerogels. This marked improvement may be attributed to the reduction of interlayer coupling in the TMD’s accompanying sol-gel processing, as previously shown in Raman analysis. In addition, the enhanced volumetric capacitance of the TMD-loaded aerogels suggests that they represent promising, scalable materials for high-density supercapacitor applications, such as hybrid vehicles or portable electronics where space is constrained^1,2,5,46^. It is worth noting that all of our 2H TMD-loaded aerogel devices perform markedly better than devices based on pure, bulk 2H TMD (2–3 F g^−1^ to 40 F g^−1^) and similarly to devices based on the 1T metallic phase of MoS_2_ (~80 F g^−1^) without pyrophoric materials^19,47^.

The addition of TMDs also improved the rate performance of the aerogels. At the highest tested current, the specific capacitance of WA-8.6 was 24% of its maximum value, compared to 15% for RFA. However, WA-17, WA-34, and MA-17 exhibited more severe drop-offs than RFA. We hypothesize that the poorer capacitance retention of these samples, as well as RFA, is correlated with their higher charge transfer resistance, a value that is measured from impedance spectroscopy, as discussed below. In addition, WA-17 exhibited excellent cycling stability, and increased in performance during repeated charging and discharging (Supplementary Figure S6). The WA-17-specific capacitance more than doubled between cycles 200 and 400, remaining 33% higher than its initial value at the final tested discharge. This enhanced capacitance during cycling may be due to additional exfoliation of the TMD or increased pseudocapacitance during charging and discharging, as observed by Bissett *et al.* for MoS_2_-graphene composite electrodes^48^.

We model the experimental EIS data, shown as Nyquist plots in Figure 6b, Supplementary Figures S4a and b, with the equivalent circuit in Figure 6a, which consists of an equivalent series resistance *R*_ESR_ followed by a constant phase element *Q* in parallel to a charge transfer resistance *R*_CT_ and a finite linear diffusion element *M*_*a*_. *R*_ESR_ comprises the resistances associated with the bulk electrolyte, bulk electrode, and ‘external’ parts of the system such as the current collector, terminals, and leads. It is represented in the Nyquist plot by the intercept of the curve with the real impedance axis. *R*_CT_ comprises the resistances due to electron transfer at interfaces in the device and specific adsorption of ions onto the active material, and is measured as the diameter of the best-fit semicircle at mid to high frequencies. Although *R*_CT_ is typically associated with the kinetics of Faradaic reactions at the electrode-electrolyte interface, the lack of peaks or troughs in the CV sweeps (Figure 6c), as well as the lack of voltage plateaus in the galvanostatic discharge profiles (Supplementary Figure S3), suggest that no such reactions occur under our testing conditions. The constant phase element (CPE) accounts for frequency dispersion of capacitance that arises from the inhomogeneities of porous and rough electrodes^49^. This causes a slight depression and angling of the semicircular arc that is characteristic of an R|C component. Finally, *M*_a_ is a particular mass-transport impedance where the diffusion layer has a finite thickness and a reflecting (non-permeable) boundary condition. This accounts for the resistance of electrolyte in pores and interfacial double-layer capacitance along pore walls^50^. In the Nyquist plot, *M*_a_ manifests as the kinked line following the semicircle, which deviates from the vertical line of an ideal capacitor.

The Nyquist plots show that *R*_CT_ increases with WS_2_ loading, and that *R*_CT_ is much greater for MA-17 than WA-17 (~21 Ω vs. 3.9 Ω), despite the former having larger pore sizes, which would reduce ion transport resistance. We hypothesize that these trends in *R*_CT_ are related to the formation of Schottky barriers between semiconducting TMDs and metals^51^. Zhang *et al.*^52^ have observed the analogous formation of a Schottky junction at the interface of MoS_2_ with sp^2^ hybridized carbon in graphite, which they attributed to the existence of metallic edge states in MoS_2_ nanosheets. Fermi level pinning may be exacerbated in these devices because the aerogels are not composed of pristine graphite, but carbonized RF polymer, whose highly defective structure hosts many charge trapping sites. In addition, while WS_2_ and MoS_2_ have similar bulk contact resistances, the incorporation of WS_2_ likely does not impact the overall charge transfer resistance of the aerogel as severely as MoS_2_ due to differences in molar loading (Table 1). Alternatively, *R*_CT_ is associated with ion adsorption within the pores of the active material^53^. On this subject, an EIS study by Bissett *et al.*^47^ on supercapacitors with exfoliated TMD membrane electrodes in aqueous Na_2_SO_4_ electrolyte, showed that the ion adsorption in MoS_2_ occurs on a much slower timescale compared to WS_2_. It is worth noting that *R*_CT_ values comparable to ours have been reported previously for coin cells containing bulk (2H phase) MoS_2_ as the active material^54^.

Interestingly, *R*_CT_ of the RFA control is similar to WA-34 (5.7 Ω). This observation is consistent with previous studies of TMD-carbon composite supercapacitors where the addition of the TMD lowered *R*_CT_ from that of the plain carbon as well as the bulk TMD^55,56^. Like WA-34 and MA-17, the RFA has larger pores which would lower ionic resistance, but no TMD’s to contribute to contact resistance within the electrode. In this case, we hypothesize that the larger *R*_CT_ is related to the significantly higher specific surface area (776 m^2^ g^−1^) and lower bulk density (0.33 g cm^−3^) of the RFA due to the absence of TMD’s, resulting in a more sparse 3D network of active material with poorer electronic conductivity whose effect is great enough to counteract the easier ion movement. In support of this claim, Yang *et al.* recently conducted a comprehensive study of pyrolyzed RF aerogel supercapacitors where the pore size was tuned by catalyst concentration^57^. They found that charge transfer resistance tended to increase in tandem with pore size and confirmed the high electronic resistance of samples with large pores by four-point probe measurements.

In contrast to *R*_CT_, *R*_ESR_ is similar for all tested aerogels, ranging from 0.56–0.76 Ω. This is not surprising given the identical composition of the pyrolyzed RF matrix and identical construction of the coin cell devices for all samples. The slight increase in *R*_ESR_ for MA-17 compared to WA-17 reflects the difference in the electrical conductivity of the constituent bulk TMDs—0.9 S cm^−1^ for WS_2_ versus 0.2 S cm^−1^ for MoS_2_ at 300 K (Refs. 22,58). Mechanical integrity may also be responsible for the differences in series resistance, as the MA electrode amalgam had a stronger tendency to crack and flake apart during the flattening process, presumably due to the weak interlayer bonding of the TMD and its higher molar loading compared to WA-17.

Another performance metric is the knee frequency *f*_k_, which is the frequency at which the semicircle transitions into the sloped linear region in the Nyquist plot, corresponding to a local minimum of the phase angle. Physically, the knee frequency signifies the point below which ions can penetrate more easily into pores of the active material, covering its entire surface to produce capacitive behavior. Within the WS_2_ aerogels, *f*_k_ increases with decreasing TMD loading—20, 28, and 54 Hz for WA-34, WA-17, and WA-8.6 respectively—although it drops to 14 Hz for the unloaded RFA. The larger charge transfer resistance of WA-34 and RFA likely accounts for the lower *f*_k_, even though they have larger pores on average compared to the other samples, which would suggest less hindrance to ion diffusion within the electrode^59^. In fact, the very short 45°-sloped Warburg region preceding the steeper part of the line in these samples also suggests lower resistance to ion diffusion in the pores. The much lower *f*_k_ of MA-17 (4 Hz) compared to WA-17 is indicative of the former’s much higher charge transfer resistance as well.

The wider pore size distribution of WA-34 and RFA also explains why these samples exhibit smaller slopes—corresponding to a lower phase angle—in the low-frequency region. With variation in pore sizes, the AC signal does not penetrate equally at a given frequency, since it is easier for ions to access larger pores than smaller pores, resulting in a shift from the theoretical vertical line of a capacitor. Song *et al.* developed a model to describe this particular frequency dispersion using a dimensionless frequency-dependent ‘penetrability’ and a pore size distribution function, showing that the slope of the line in the Nyquist plot decreases for pore distributions with greater standard deviation^60,61^.

The current–voltage plots from the 20 mV s^−1^ CV sweeps are shown in Figure 6c and Supplementary Figure S4c. While an ideal capacitor exhibits a rectangular shape, the voltammograms of the aerogel samples exhibit rounded corners, indicating resistance to ion diffusion that slows the response of the current to changes in the direction of the voltage sweep. In agreement with EIS, the MA-17 shows the largest ion diffusion resistance, represented by a lens-shaped voltammogram. Similarly, WA-34 and RFA also have significantly distorted CV curves, while WA-8.6, and WA-17 have the most rectangular curves. The more rectangular CV shape of WA-8.6 and WA-17 is also corroborated by their higher knee frequencies compared to the other two samples. The lack of peaks and troughs in the voltammograms of the three aerogels indicate that no redox reactions occur over the tested voltage range and that the mechanism of capacitance is purely double layer. While Na^+^ ions are known to intercalate between the layers of TMD particles, they are unlikely to do so except at extremely low scan rates^62,63^.

## Conclusion

In conclusion, we have demonstrated a rapid, scalable nanomanufacturing process for the production of TMD-doped carbon aerogel composites via polycondensation of resorcinol and formaldehyde catalyzed with hydrochloric acid in acetonitrile. Compared to typical aerogel processing (24 h), the reaction presented here occurs in 2% of the time without sacrificing the narrow pore sizes or high surface areas of a standard RF aerogel. This synthesis outlines a general method to support TMD’s with high electrical conductivity and porosity which is applicable to other stable TMD’s. Any advances in TMD synthesis or exfoliation can be directly incorporated via this process. Given the wide potential range of TMD applications, including electrochemical, photovoltaic, and catalytic, this rapid synthesis will accelerate combinatorial optimization of design parameters to engineer new devices. As a proof of concept, we explored the performance of MoS_2_ and WS_2_-doped carbon aerogels as electrodes for supercapacitors. An initial screening of device performances indicates that the addition of TMD’s yields electrodes that are cyclically stable and offer volumetric capacitances up to 127% higher than pyrolyzed RF alone. Further, the ability to rapidly process new materials into composites is magnified by the range of applications for high surface area, conductive supports, and we believe this scalable manufacturing methodology will find widespread use.

## Figures and Tables

**Table 1:** Summary of pyrolyzed TMD aerogel composite properties

Property	Aerogel sample				
	MoS_2_17 mg mL^−1^	WS_2_8.6 mg mL^−1^	WS_2_17 mg mL^−1^	WS_2_34 mg mL^−1^	No loading
TMD Mole %	6.70%	2.30%	4.40%	8.40%	0.00%
XRD crystallite Thickness (nm)*	64	99	104	95	—
BET surface area (m^2^ g^−1^)	408	500	620	514	776
Gravimetric capacitance (F g^−1^)†	84.5	60.3	56.2	64.7	87.5
Volumetric capacitance (F cm^−3^)	52.5	54.2	50.9	59.8	26.3
Molar capacitance (F mol^−1^)	1650	970	1110	1760	1060
Equivalent series resistance *R*_ESR_ (Ω)	0.76	0.60	0.68	0.56	0.60
Charge transfer resistance *R*_CT_ (Ω)	21	2.6	3.9	5.7	5.7
Knee frequency *f*_k_ (Hz)	4	54	28	20	14

* in the (002) plane.

† Capacitance values from galvanostatic discharge profile at lowest tested current density.
