# Supplementary material for: Rapid synthesis of transition metal dichalcogenide–carbon aerogel composites for supercapacitor electrodes
Source: Microsyst Nanoeng. 2017 Jul 17;3:17032. doi: 10.1038/micronano.2017.32 (PMC6444994; doi:10.1038/micronano.2017.32)
Supplement: Supplementary Information [file micronano201732-s1.pdf]

Supplementary file

Rapid synthesis of transition metal dichalcogenide–carbon aerogel composites for supercapacitor electrodes

Matthew J. Crane[1,*], Matthew B. Lim[2,*], Xuezhe Zhou[2] and Peter J. Pauzauskie[2,3]

Microsystems & Nanoengineering (2017) **3,** 17032; doi:10.1038/micronano.2017.32; Published online: 17 July 2017

1. THEORETICAL SURFACE AREA CALCULATION

For each TMD, we calculated the theoretical surface area by extrapolating from the unit cell and neglecting edge effects. As an example calculation for WS_2, the specific surface area is

$$\frac{a^2 N_a}{MW} \tag{1}$$

where a is the (100) lattice parameter (a = b = 3.1532 Å and c = 12.3230 Å), MW is the molecular weight of the TMD unit cell, and N_a is Avogadro's number. For WS_2, MoS_2, and $NbSe_2$, the theoretical specific surface areas are 482.77, 752.14, and 468.87 m^2/g, respectively.

2. SCHERRER ANALYSIS OF TMD-LOADED AEROGELS

We quantified the size of the TMD crystals loaded into the aerogels using the Scherrer equation,

$$L_a = \frac{1.32\lambda_{K\alpha}}{\beta cos\theta} \tag{2}$$

where the crystal thickness L_a in the (002) direction is a function of the x-ray wavelength $\lambda_{K\alpha}$, the position of the (002) diffraction peak θ, and the full width at half maximum (FWHM) of the peak β, which we correct for instrument broadening with $\beta = \sqrt{\beta_m^2 \quad \beta_{ref}^2}$, where β_m and β_{ref} are the measured FWHM of the TMD (002) peak and the measured FWHM of a peak of a corundum standard that occurs at a similar Bragg angle to the TMD (002) peak[1].

3. VOLUMETRIC CAPACITANCE CALCULATION FROM GALVANOSTATIC DISCHARGE

We calculated volumetric capacitance of a single electrode from galvanostatic discharge profiles using the equation adapted from gravimetric capacitance[2]:

$$C_{s,v,galv} = \frac{8\rho I \int_{t_1}^{t_2} V dt}{m_a (V_2 \quad V_1)^2} \tag{3}$$

where I is the constant discharge current, m_a is the total mass of active material in both electrodes, ρ is the bulk density of the active material, and (t_1, V_1), (t_2, V_2) are two chosen points on the voltage-time curve. Our reported values use the full potential window of our discharge tests (0.9–0.1 V).

We note that, while gravimetric capacitance is frequently used as a figure of merit for supercapacitors, volumetric capacitance represents a more pragmatic metric for high density energy storage and a more reliable parameter in evaluating the charge-storage performance of low bulk density electrode materials, like aerogels[3,4]. Additionally, volumetric capacitance normalizes results for significant changes in density. Finally, many earlier studies on the supercapacitor applications of TMD-carbon composites employed thin film-like electrodes obtained through filtration[5–8]. Owing to the very small mass of active material, this can lead to artificially high values of gravimetric capacitance that do not translate to larger-scale devices.

4. VOLUMETRIC CAPACITANCE CALCULATION FROM EIS

Volumetric capacitance can also be calculated from electrochemical impedance spectroscopy (EIS) using the equation[9]:

$$C_{s,v,EIS} = \frac{\rho}{2\pi f Z'' m_a} \tag{4}$$

where f is the frequency and Z" is the imaginary component of impedance, m_a is the total mass of active material in both electrodes, and ρ is the bulk density of the active material.

Supplementary Figure S5 plots the EIS-derived volumetric capacitances of the aerogels as a function of frequency. Even at the lowest frequency of 10 mHz where volumetric capacitance is maximized, it is less than one-third of the maximum value obtained from galvanostatic testing. We note that the relatively low capacitance obtained from EIS versus other methods is well-documented for supercapacitors based on porous carbons[9–12]. For example, Lufrano *et al.* observed that the capacitance of carbon composite electrodes with H_2SO_4 electrolyte was 20% lower when measured with EIS than with galvanostatic charge-discharge tests[9]. Various explanations have been proposed for the discrepancy—such as the increased hindrance for alternating current penetration into the bulk electrode[10,11]; or the presence of deeply trapped ions that are immobile under AC conditions but can be released at low potentials in DC methods[13]. However, nothing conclusive has emerged to date.

REFERENCES

1 Berhault G, Perez De la Rosa M, Mehta A *et al.* The single-layered morphology of supported MoS2-based catalysts—The role of the cobalt promoter and its effects in the hydrodesulfurization of dibenzothiophene. *Appl Catal Gen* 2008; **345**: 80–88.

2 Roldán S, Barreda D, Granda M *et al.* An approach to classification and capacitance expressions in electrochemical capacitors technology. *Phys Chem Chem Phys* 2015; **17**: 1084–1092.

[1]Department of Chemical Engineering, University of Washington, Seattle, WA 98195-1750, USA; [2]Department of Materials Science & Engineering, University of Washington, Seattle, WA 98195-2120, USA and [3]Fundamental & Computational Sciences Directorate, Pacific Northwest National Laboratory, Richland, WA 99352, USA.
Correspondence: Peter J. Pauzauskie, (peterpz@uw.edu)
*These authors contributed equally to this work.

3 Gogotsi Y, Simon P. True Performance Metrics in Electrochemical Energy Storage. *Science* 2011; **334**: 917–918.
4 Wang Q, Yan J, Fan Z. Carbon materials for high volumetric performance supercapacitors: design, progress, challenges and opportunities. *Energy Environ Sci* 2016; **9**: 729–762.
5 Liu Y, Wang W, Huang H *et al*. The highly enhanced performance of lamellar WS 2 nanosheet electrodes upon intercalation of single-walled carbon nanotubes for supercapacitors and lithium ions batteries. *Chem Commun* 2014; **50**: 4485–4488.
6 Ratha S, Rout CS. Supercapacitor Electrodes Based on Layered Tungsten Disulfide-Reduced Graphene Oxide Hybrids Synthesized by a Facile Hydrothermal Method. *ACS Appl Mater Interfaces* 2013; **5**: 11427–11433.
7 Shang X, Chi J-Q, Lu S-S *et al*. Carbon fiber cloth supported interwoven WS2 nanosplates with highly enhanced performances for supercapacitors. *Appl Surf Sci* 2017; **392**: 708–714.
8 Huang K-J, Wang L, Zhang J-Z *et al*. Synthesis of molybdenum disulfide/carbon aerogel composites for supercapacitors electrode material application. *J Electroanal Chem* 2015; **752**: 33–40.
9 Lufrano F, Staiti P, Minutoli M. Evaluation of nafion based double layer capacitors by electrochemical impedance spectroscopy. *J Power Sources* 2003; **124**: 314–320.
10 Fuertes AB, Lota G, Centeno TA *et al*. Templated mesoporous carbons for supercapacitor application. *Electrochimica Acta* 2005; **50**: 2799–2805.
11 Xing W, Qiao SZ, Ding RG *et al*. Superior electric double layer capacitors using ordered mesoporous carbons. *Carbon* 2006; **44**: 216–224.
12 K. Kampouris D, Ji X, P. Randviir E *et al*. A new approach for the improved interpretation of capacitance measurements for materials utilised in energy storage. *RSC Adv* 2015; **5**: 12782–12791.
13 Tanguy J, Mermilliod N, Hoclet M. Capacitive Charge and Noncapacitive Charge in Conducting Polymer Electrodes. *J Electrochem Soc* 1987; **134**: 795–802.
14 Bisquert J, Randriamahazaka H, Garcia-Belmonte G. Inductive behaviour by charge-transfer and relaxation in solid-state electrochemistry. *Electrochimica Acta* 2005; **51**: 627–640.

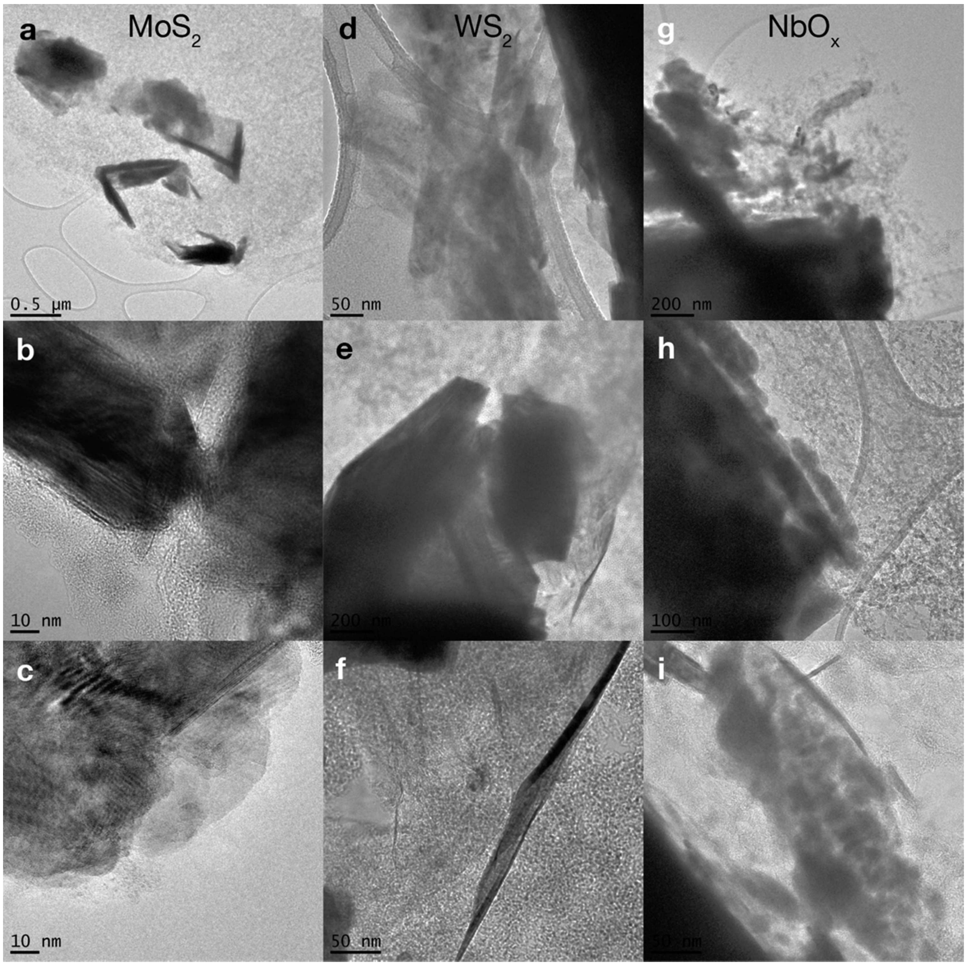

Figure S1 Additional TEM images of pyrolyzed MoS_2 (**a**–**c**), WS_2 (**d**–**f**), and NbO_x (**g**–**i**) loaded aerogels showing exfoliation and morphology of TMD sheets within the RF support matrix. Samples pictured were synthesized from 17 mg ml^{-1} TMD dispersions.

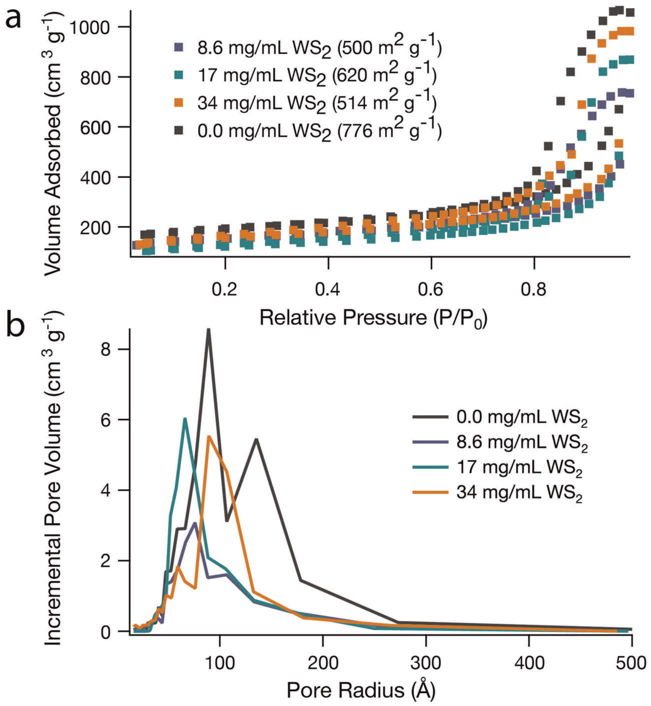

Figure S2 Nitrogen sorption isotherms with BET surface area (**a**) and BJH pore size distribution (**b**) of pyrolyzed aerogel with different mass loadings of WS_2.

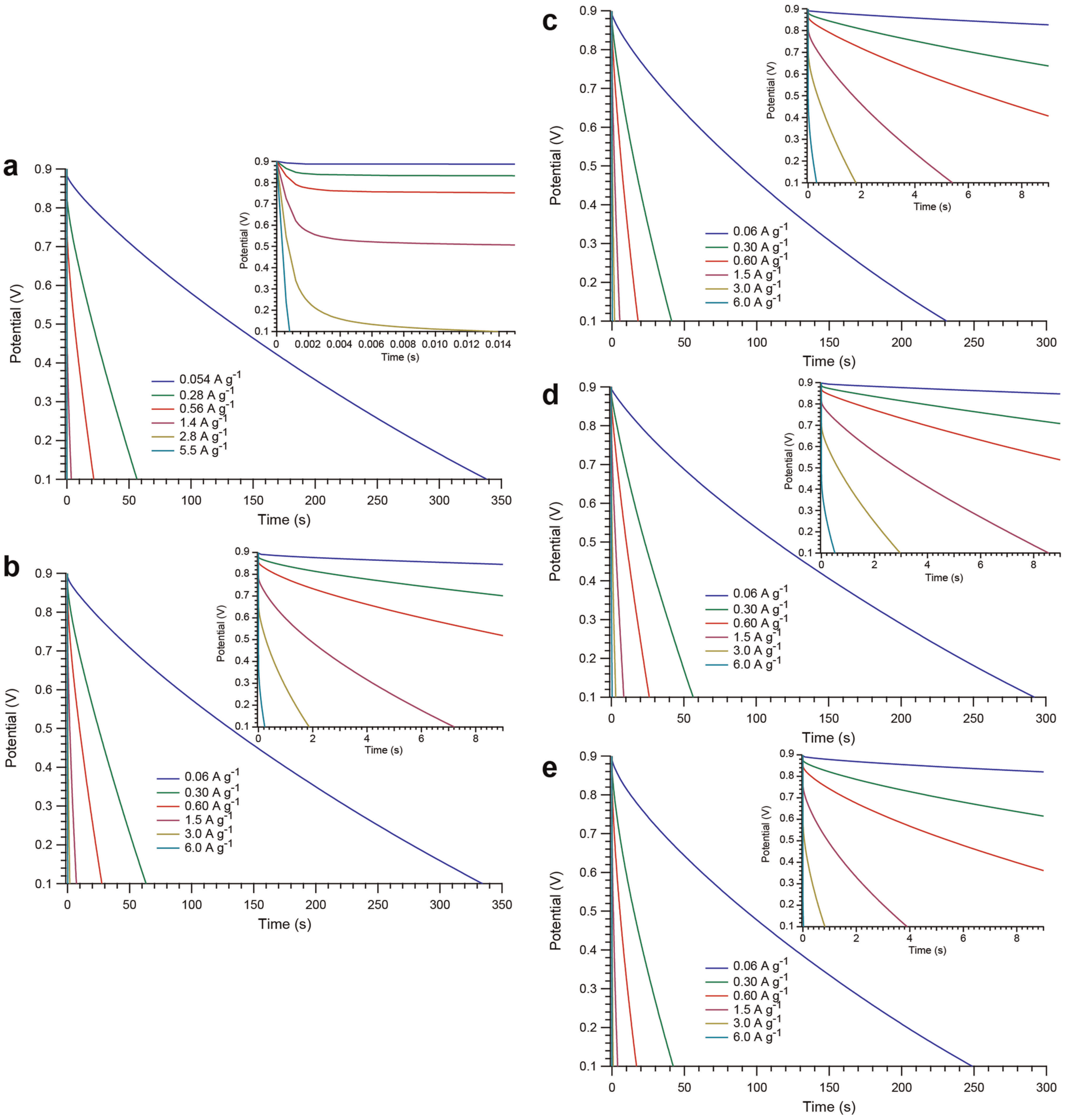

Figure S3 Galvanostatic discharge curves, used to calculate specific capacitance values, for supercapacitors containing MA-17 (**a**), RFA (**b**), WA-8.6 (**c**), WA-17 (**d**), and WA-34 (**e**) aerogel electrodes. Insets show same curves at shorter timescales.

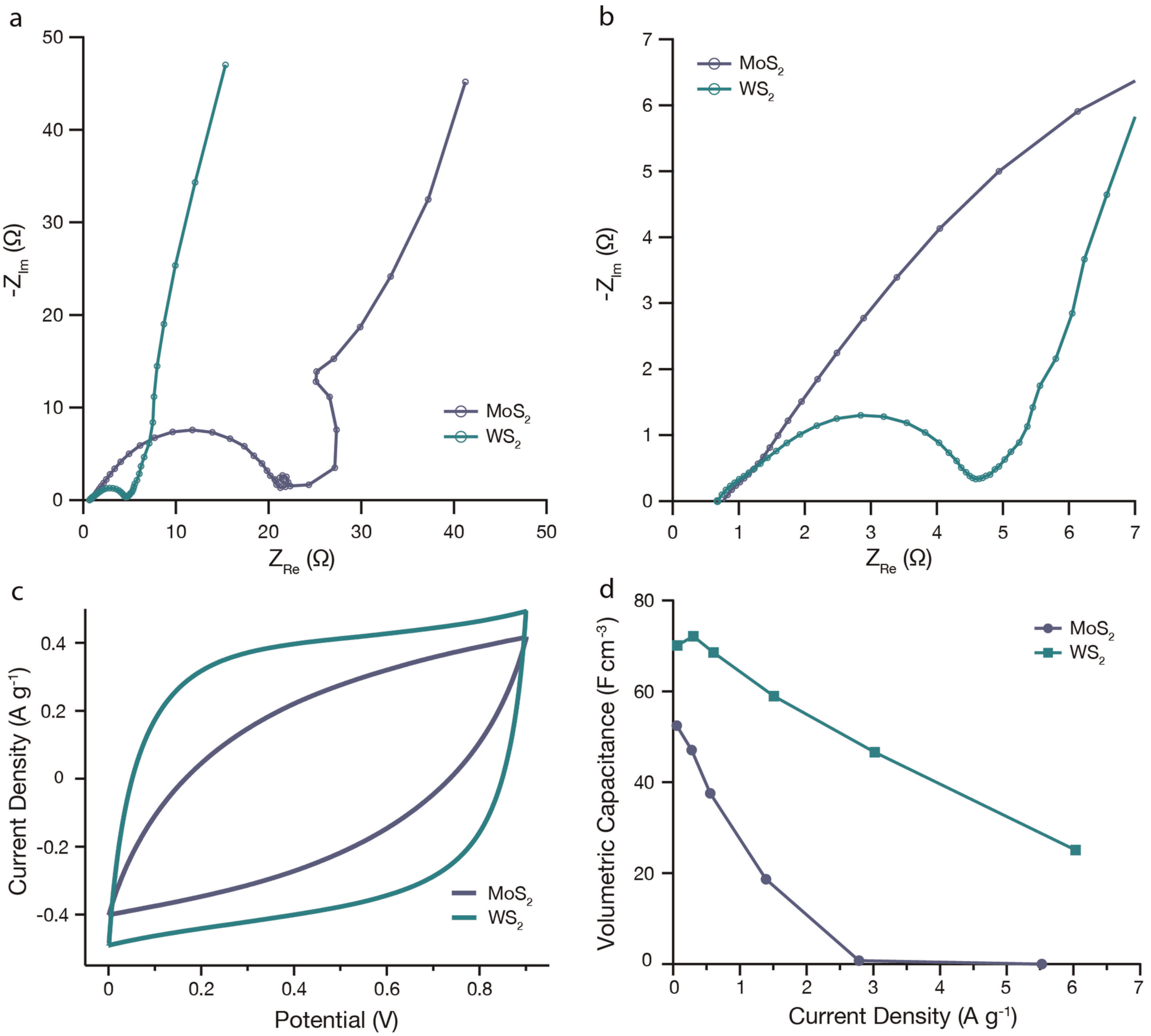

Figure S4 Electrochemical characterization of MA-17 and WA-17 supercapacitors. This includes Nyquist plots from EIS (**a**: full range, **b**: detail of high to mid frequency), cyclic voltammetry at a sweep rate of 20 mV s^{-1} (**c**), and the specific volumetric capacitance (**d**) as a function of applied current density from galvanostatic tests. *Note on Figure S4.* The Nyquist plot of MA-17 exhibits unusual characteristics at lower frequencies that are difficult to interpret. Between the semicircular R|C region and the steeply sloped constant phase region, there is a small loop followed by a larger arc that curves back on the real impedance axis, instead of the expected 45° Warburg line. These features indicate low-frequency inductive behavior in the system, which is not typically observed in supercapacitors. We note, however, that these features also appeared for duplicate coin cells made with the same active material. One possible explanation is offered by Bisquert *et al.*, who extensively studied inductive phenomena in the context of a porous, heterogeneous electroactive material composed of two different solids in contact with electrolyte[14]—a good model for our TMD/carbon aerogel composite electrodes. They found that inductive behavior resulted from the coupled dielectric relaxation in the two solid phases, with the relaxation being driven by changes in the electrochemical potential of the phases due to charge transfer between them.

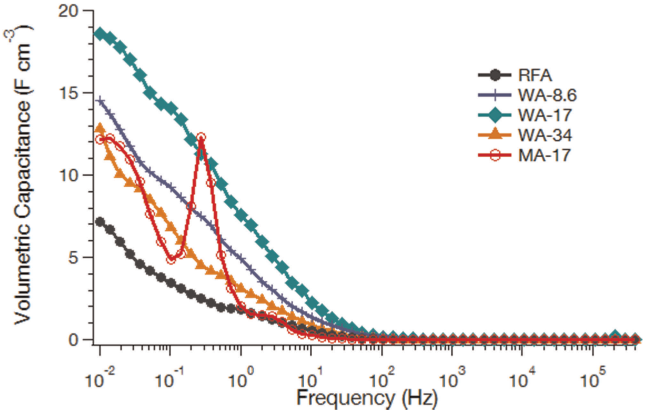

Figure S5 Specific volumetric capacitance, derived from electrochemical impedance spectroscopy, as a function of frequency for the different aerogel supercapacitors.

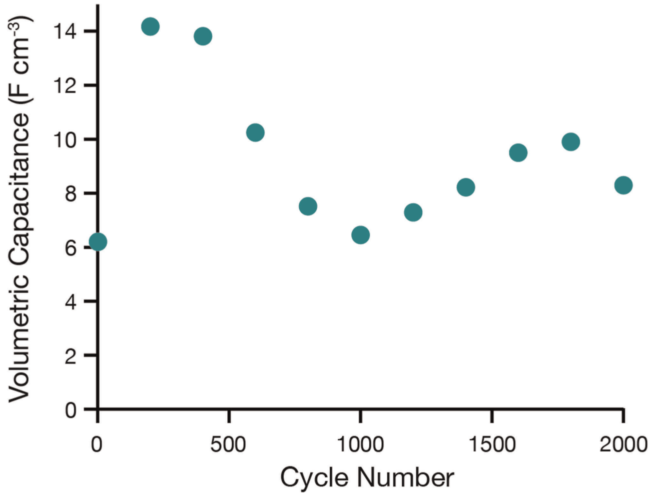

Figure S6 Specific volumetric capacitance of WA-17 during galvanostatic cycling at 0.25 A g^{-1}, derived from discharge curves at selected cycles.

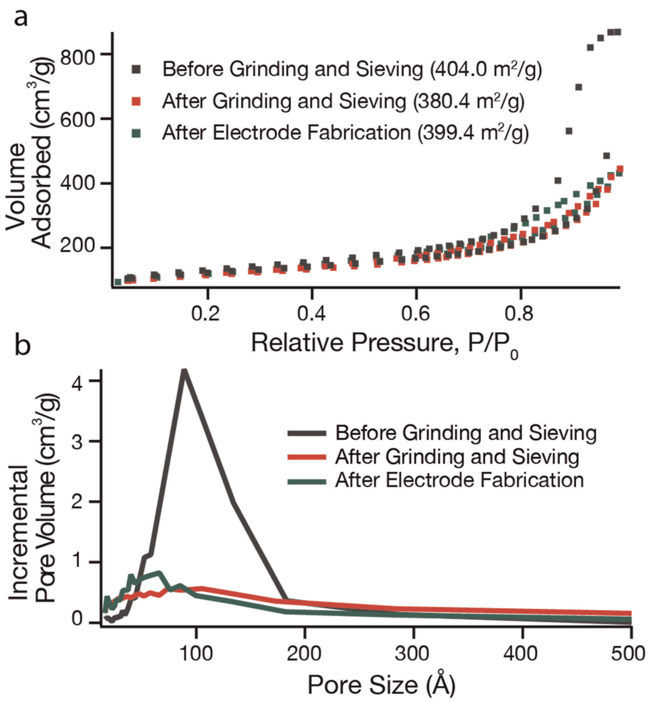

Figure S7 Nitrogen sorption isotherms with BET surface area (**a**) and BJH pore size distribution (**b**) of pyrolyzed WS_2-loaded aerogel before processing, after grinding and sieving, and after forming into a supercapacitor electrode with PTFE binder and carbon black.

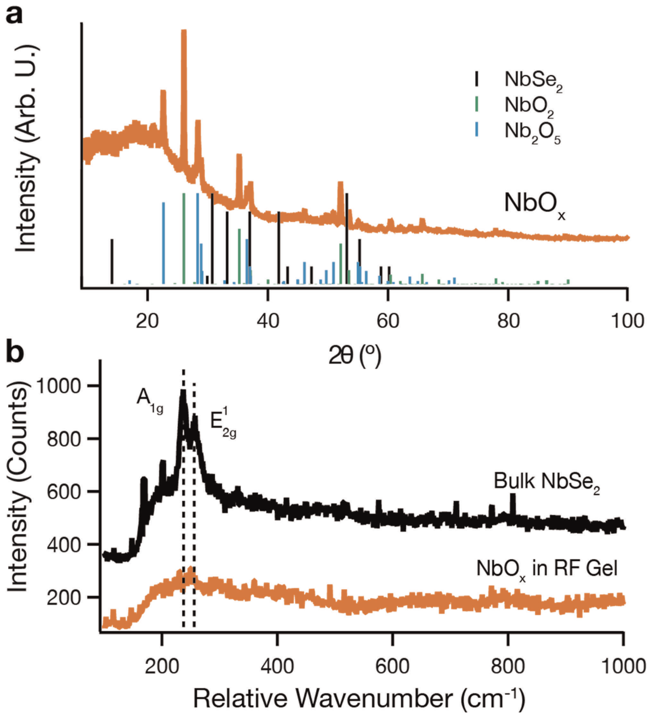

Figure S8 Raman (**a**) and XRD (**b**) characterization of the NbSe2-loaded aerogel after pyrolysis at 800 °C.

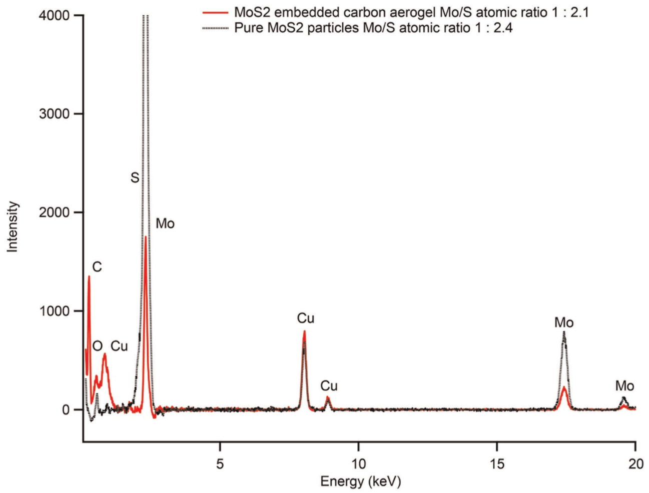

Figure S9 Energy dispersive x-ray spectroscopy of MoS_2 powder and MoS_2-loaded aerogel after pyrolysis.

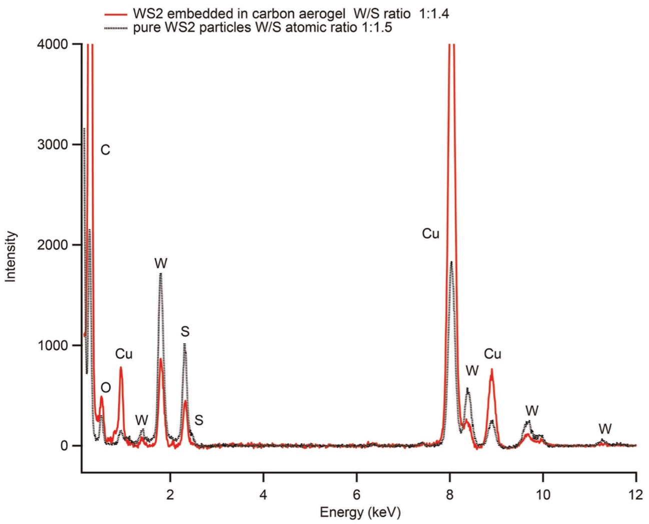

Figure S10 Energy dispersive x-ray spectroscopy of WS_2 powder and WS_2-loaded aerogel after pyrolysis.
